# A postoperative brain abscess due to Propionibacterium acnes in an immunocompetent adult with incidental discovery

**DOI:** 10.1016/j.amsu.2022.104451

**Published:** 2022-08-19

**Authors:** Mehdi Borni, Souhir Abdelmouleh, Haifa Mechergui, Emna Elouni, Mohamed Zaher Boudawara

**Affiliations:** Department of Neurosurgery – UHC Habib Bourguiba –Sfax, Tunisia

**Keywords:** Propionibacterium acnes, Brain abscess, Antibiotic therapy, Surgery

## Abstract

**Introduction:**

and importance: Propionibacterium acnes (P. acnes) is an anaerobic, lipophilic, Gram-positive bacteria of the commensal skin flora. It may also be present on the mouth's mucosa, nose, urogenital tract, and large bowel. P. acnes is an unusual and rare agent of intracerebral abscess although in recent years some publications suggest that its frequency in brain surgery is increasing.

**Case presentation:**

The authors report a case of an incidental cerebral abscess during follow-up imaging in a 63-year-old male patient operated on twice for cerebral meningioma the last of which was 3 months ago without placement of any surgical implant with uneventful postoperative course.

**Clinical discussion:**

P. acnes is still an sunder-appreciated cause of post-neurosurgical infection. Time between neurosurgery and infection is variable ranging from few months to many years. Its culture time is long, with currently an average time to positivity of six days (2–15 days), justifying prolonged cultures.

**Conclusion:**

Intracranial infections by P. acnes are not quite frequent. We emphasize the need to send samples for culture of anaerobes in this type of complications before giving a negative result.

## Highlights

• Propionibacterium acnes (P. acnes) is an anaerobic, lipophilic, Gram-positive bacteria belonging to the Propionibacterium spp family.
• It is a bacterium of the commensal skin flora, where it colonizes the hair follicles and the sebaceous glands.
• P. acnes is an unusual and rare agent of intracerebral abscess, although in recent years some publications suggest that its frequency in brain surgery is increasing.
• Culture time is long, with currently an average time to positivity of six days (2–15 days), justifying prolonged cultures.
• Concerning the principles of treatment, it is uncommon to be certain of the diagnosis of P. acnes infection.
• This current case is an addition to the literature which remains rare demonstrating that this bacterium is able to cause CNS damage in immunocompetent subjects and must be taken into account in the differential for the cause of intracranial suppuration.

## 1 Introduction

Propionibacterium acnes (P. acnes) is an anaerobic, lipophilic, Gram-positive bacteria belonging to the Propionibacterium spp family. It is a bacterium of the commensal skin flora, where it colonizes the hair follicles and the sebaceous glands. It may also be present on the mouth's mucosa, nose, urogenital tract, and large bowel [1]. Its pathogenic role has been demonstrated in multiple infections: cutaneous, digestive, cardiovascular, but also in orthopedic surgery [[1], [2], [3]].

P. acnes is an unusual and rare agent of intracerebral abscess [4], although in recent years some publications suggest that its frequency in brain surgery is increasing. The risk factors for developing such abscesses are mostly related to brain surgical implants and usually have a mild clinical course, where symptoms are nonspecific [5,6].

The authors report a case of an incidental cerebral abscess during follow-up imaging in a 63-year-old male patient operated on twice for cerebral meningioma the last of which was 3 months ago without placement of any surgical implant with uneventful postoperative course.

This case report has been reported in line with the SCARE Criteria [7].

## 2 Case presentation

A 63-year-old immunocompetent male patient with prior twice resection of a frontal atypical meningioma (central nervous system world health organization grade 2) the last of which was 3 months ago before presentation, was admitted to our department of neurosurgery from the outpatient clinic of our hospital for incidental discovery of a brain abscess on the routine postoperative enhanced magnetic resonance imaging (MRI). During the interview with the patient, he reported no recent clinical symptoms and he insisted that his postoperative course was strictly free of abnormalities. Throughout this period, he denied any infection of the surgical wound and that the stitches were removed without incident 15 days postoperatively according to conventional methods. Upon examination, the patient was awake with good orientation to time, place, and person without any neurological motor or sensory deficit. His surgical wound was strictly clean and dry without any pus or serous fluid discharge. He was in apyrexia and good general condition. Initial vital signs were as follows: blood pressure, 130/82 mmHg; pulse rate, 60/min; respiratory rate, 20/min.

**Fig. 1:**
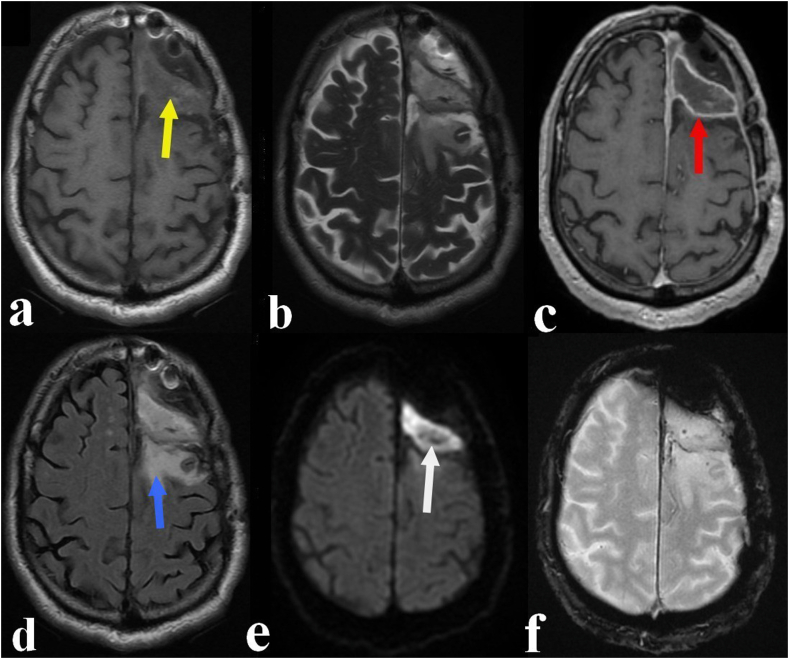
Axial brain enhanced MRI revealing a left frontal fluid collection at the site of the measuring 65 × 69 × 70 mm in diameter. This collection appeared in isosignal on T1-weighted sequence (**a**; yellow arrow) and discrete hypersignal on T2-weighted sequence (**b**) with heterogeneous and peripheral enhancement after gadolinium chelate injection (**c**; red arrow). On T2 FLAIR sequence (**d**; blue arrow), it was surrounded by a perilesional edema with a significant hypersignal on DWI image (**e**; white arrow). There was no bleeding on the gradient echo GRE sequence (**f**). (For interpretation of the references to colour in this figure legend, the reader is referred to the Web version of this article.)

The MRI he brought (Fig. 1) revealed a left frontal fluid collection at the site of the old meningioma measuring 65 × 69 × 70 mm in diameter. This collection was in isosignal on T1-weighted sequence and discrete hypersignal on T2-weighted sequence with heterogeneous and peripheral enhancement after gadolinium chelate injection. On T2 fluid attenuated inversion recovery image (FLAIR), it was surrounded by a perilesional edema with a significant hypersignal on diffusion weighted sequence (DWI) and without bleeding on the gradient echo sequence (GRE). All these radiological features suggested a brain abscess.

A complete assessment of the blood test revealed a normal white blood cell count of 7800 WBCs/μl (neutrophils, 54%; lymphocytes, 33%; monocytes, 8%; eosinophils, 4%; and basophils, 1%) with a C-reactive protein level below 3. The rest of the blood test was within reference ranges. The frontal chest x-ray was also normal.

An exhaustive toxicology and infectious disease investigation, including peripheral blood cultures, was conducted. These toxicological results were not revealing and proved negative for HIV, syphilis, hepatitis B and hepatitis C.

Informed consent was obtained from the patient and a routine preanaesthetic testing was performed urgently before surgery. The patient underwent, therefore, a complete evacuation of the collection, via his old craniotomy, having liquefied yellowish pus with large amounts of purulent matter with pasty consistency. The postoperative course was marked by the onset of a grade 4/5 left hemiparesis. The patient was empirically started on cefotaxime (200 mg/kg/day intravenous divided every 6 hours), metronidazole (15 mg/kg/day IV divided every 8 hours), and vancomycin (60 mg/kg by slow intravenous injection). All samples taken were transferred into several 10 ml sterile syringe and sent immediately to the microbiology laboratory. Microscopic direct examination showed an abundant inflammatory reaction and Gram staining revealed the presence of immobile bifid and not sporulated pleomorphic gram-positive bacilli. The samples were cultured on blood, chocolate and thioglycolate broth agar plates, incubated aerobically for 10 days at 37 °C, no microbial development was observed on the blood and chocolate agar plates. After a week of incubation, turbidity of the liquid medium was observed and Gram staining showed pleomorphic gram-positive bacilli that did not develop in aerobiosis concluding to a multi-resistant agent: Propionibacterium acnes. Later, the antibiogram study (Table 1) showed a significant sensitivity to several antibiotics including cefotaxime and vancomycin and a resistance for both kanamycin and gentamicin (minimum inhibitory concentration range respectively 2–8 and 2–4).

**Table 1:** Antibiotic susceptibility pattern of Propionibacterium acnes isolated from brain abscess.

Antibiotic	Interpreted result	Diam	Diam Range	MIC	MIC Range
Amoxicillin 20 μg	Sensitive		0–0		0–0
Amoxicillin - Clavulanite acid 20-10 μg	Sensitive		17–21		4–8
Imipenem 10 μg	Sensitive		17–24		2–8
Kanamycin 30 μg	Resistant		15–17		8–16
Rifampicin 5 μg	Sensitive		0–0		0–0
Vancomycin	Sensitive		10–10		0–0
Ciprofloxacin 5 μg	Sensitive		0–0		0.25–0.5
Rifampicin 30 μg	Sensitive	46	14–19	<0.0156	4–16
Trimethoprim + Sulfamides 1.25–23.75 μg	Sensitive	52	10–16	<0.0156	2–8
Gentamicin 10 μg	Resistant		16–18		2–4
Tetracyclin 30 μg	Sensitive		0–0		0–0

**MIC**: minimum inhibitory concentration; **Diam**: diameter.

The patient has completed 30 days of intravenous antibiotic therapy (cefotaxime and vancomycin). He continued, therefore, to improve with complete disappearance of hemiparesis and no focal findings on examination. The final brain computed tomography revealed no signs of suppuration. The antibiotic therapy was then switched to the oral route made of ciprofloxacin (1000 mg/day divided every 12 hours) and sulfamethoxazole/trimethoprim (2400 mg divided every 8 hours) for a period of 15 days. The patient was, therefore, discharged from our department with an outpatient clinic appointment.

## 3 Clinical discussion

Propionibacterium acnes is a gram-positive, non-spore-forming, anaerobic bacillus that is part of normal skin's flora, mucous membranes, nasopharynx, oral cavity, intestinal and genitourinary tracts. It is considered to have a low level of pathogenicity. Since it is a skin commensal, it may be considered a contaminant in blood cultures, wound materials, and other puncture fluids. It has been postulated as the causative agent of inflammatory acne. Several reports in the literature showed that it may cause endocarditis, discitis, osteomyelitis, and central nervous system involvement including meningitis, subdural empyema and cerebral abscesses [[8], [9], [10]]. Intracranial infections due to P. acnes are not common. In 2007, McClelland et al. [11] published a series of 1587 cranial surgeries, of which 0.8% developed postoperative infection. P. acnes was isolated in 28.6%, considered the second causative agent of brain abscess after *Staphylococcus aureus*, isolated by culture in 43% of cases. According to this study, which concerns the largest number of neurosurgical acts, the frequency of post-surgical infection is 5–6 times lower than in the other published series. In a more recent study published in 2014, Reuter et al. [12] reported a series of 125 postoperative infections. P. acnes were present in more than 50% of infections.

P. acnes is still an sunder-appreciated cause of post-neurosurgical infection [6]. Time between neurosurgery and infection is variable ranging from few months to many years. Chung et al. [4] reported a case of brain abscess caused by P. acnes 13 Months after neurosurgery that was confirmed by 16S rRNA gene sequencing. In 2009, Kranick et al. [13] reported a very delayed brain abscess 10 years postoperatively. In 2007, Nisbet et al. [6] reported a series of 28 patients who underwent neurosurgery. The mean time between surgery and infection varied between 12 and 1578 days with an average of 54 days. However, in 2022 Smith et al. [14] reported a case of spontaneous P. acnes brain abscess with intraventricular rupture without any prior neurosurgical intervention. In our case, the mean time between surgery and presentation was of 3 months which was consistent with the literature data.

In standard bacteriology, different culture media may be used, without a specific medium being superior in case of suspected infection. However, the culture time is long, with currently an average time to positivity of six days (2–15 days), justifying prolonged cultures [15,16]. In our case, we needed only 7 days of culture to make our diagnosis and recognize our bacterium.

The bacteriological analysis must be carried out from deep samples of good quality (avoiding any skin contamination), in sufficient number and sufficient volume, which excludes the use of swabs [17]. For some authors, it is possible to improve the sensitivity of bacteriological examinations by using sonication techniques of the explanted material, which “removes the bacteria present in the biofilm” [18]. However, this complex technique is not available in all laboratories. For some, grinding techniques, which are simpler to implement, are also effective [19]. We did not perform any of these techniques to make our diagnosis.

Concerning the principles of treatment, it is uncommon to be certain of the diagnosis of P. acnes infection. It is therefore imperative, apart from these rare cases or specialized teams, not to take the risk of therapeutic failure due to a diagnostic error. It is therefore necessary, in the majority of cases, to initially practice a probabilistic antibiotic therapy which will be subsequently adapted. In our patient a probabilistic intravenous antibiotic therapy made of cefotaxime, metronidazole, and vancomycin was started and maintained for 30 days and was then switched to oral ciprofloxacin and sulfamethoxazole/trimethoprim for a period of 15 added days.

The final adapted antibiotic treatment should only be implemented after receipt of all the final results of the bacteriological samples. The sole situation that potentially makes it possible to avoid the probabilistic antibiotic therapy phase is the existence preoperatively of positive results from abscess puncture of excellent technical quality. However, this situation is rare outside of certain expert centers. In our case we did not perform any puncture and we aimed the evacuation of the whole abscess through the old craniotomy.

The susceptibility of P. acnes to antibiotics remains quite good, in particular to beta-lactams, quinolones and rifampicin; nevertheless, strains resistant to certain antibiotics (clindamycin in particular) appear and we must remember the natural resistance of P. acnes to metronidazole, despite its anaerobic nature [1]. A European study with 304 P. acnes isolates from 13 laboratories in 13 different countries tested 6 antibiotics [20]. Of the isolates, 2.6% were resistant to tetracycline, 15.1% to clindamycin, and 17.1% to erythromycin, with no reported resistance to linezolid, benzathine penicillin, and vancomycin [20]. There was variation in the resistance profile between countries, with 83% in Croatia, 60% in Italy and none in Norway, and blood isolates were predominant among resistant [20]. The *P. acnes* isolate obtained from our patient was susceptible to beta-lactams, quinolone, and rifampicin but it was resistant to kanamycin and gentamicin. Susceptibility to metronidazole was not tested.

As P. acnes has low susceptibility to cephalosporins, antibiotic prophylaxis should be reviewed in surgical procedures in which this bacterium is an important source of postoperative complication [21].

Concerning the prognosis, the mortality linked to intracranial infections with P. acnes does not seem to be very present. In the literature data, rare articles have discussed the morbidity associated with focal neurological deficits after treatment. Our case, given the incidental finding, supports the possibility that with appropriate treatment and good management, even significant neurological deficits may be recovered or may disappear.

In total, the authors here report the case of a patient with an intracranial P. acnes abscess. This current case is an addition to the literature which remains rare demonstrating that this bacterium is able to cause CNS damage in immunocompetent subjects and must be taken into account in the differential for the cause of intracranial suppuration.

## 4 Conclusion

Intracranial infections by P. acnes are not quite frequent, but this agent should be considered as a potential cause of post-surgical brain abscess. We emphasize the need to send samples for culture of anaerobes in this type of complications before giving a negative result. We emphasize the relevance of the Gram stain as a rapid method to guide the diagnosis, and that the microbiology laboratory must have adequate methodology for the recovery of this type of microorganisms.

## Ethical approval

The study is exempt from ethnical in our institution.

## Sources of funding

There is no sources of funding for this research.

## Author contribution

Mehdi Borni, Souhir Abdelmouleh: study concept, data interpretation, writing the paper.

Mehdi Borni, Haifa Mechergui, Emna Elouni: writing the paper.

Mehdi Borni, Souhir Abdelmouleh: study concept.

Med Zaher Boudawara: study concept and validation.

## Consent

Written informed consent was obtained from the patient for publication of this case report and accompanying images.

## Registration of research studies

1. Name of the registry:
2. Unique identifying number or registration ID:
3. Hyperlink to your specific registration (must be publicly accessible and will be checked):

## Guarantor

Mehdi Borni:Department of Neurosurgery – UHC Habib Bourguiba –Sfax (Tunisia)Mail: borni.mehdi13@gmail.com.

## Provenance and peer review

Not commissioned, externally peer reviewed.

## Declaration of competing interest

The authors declared no potential conflicts of interests with respect to research, authorship and/or publication of the article.

## Patient perspective

During hospitalization and at the discharge, the patient was given the opportunity to share her perspective on the intervention she received and she was satisfied with the care.

## Declaration of competing interest

The authors declare that there are no conflicts of interest regarding the publication of this article.
